# Is conduction system pacing more effective than right ventricular pacing in reducing atrial high-rate episodes in patients with heart failure and preserved ejection fraction?

**DOI:** 10.3389/fphys.2024.1500159

**Published:** 2024-12-02

**Authors:** Ying Chen, Zhu-Lin Ma, Fei Liu, Nan Wang, Yue-Yang Ma, Zi-An Guan, Zhuang-Chuan Zhe, Yun-Long Xia, Ying-Xue Dong

**Affiliations:** Department of Cardiology, First Affiliated Hospital of Dalian Medical University, Dalian, China

**Keywords:** conduction system pacing, right ventricular pacing, atrial high-rate episodes, atrial fibrillation, heart failure with preserved ejection fraction

## Abstract

**Background:**

The relationship between conduction system pacing (CSP) and the incidence of atrial fibrillation (AF) in patients with heart failure and preserved ejection fraction (HFpEF) remains uncertain. This study aims to investigate the occurrence of atrial high-rate episodes (AHREs) following CSP in patients with HFpEF, in comparison to right ventricular pacing (RVP).

**Methods:**

Patients with HFpEF who received dual-chamber pacemakers for atrioventricular block were retrospectively enrolled from January 2018 to January 2023. Both new-onset and progressive AHREs were recorded, along with other clinical data, including cardiac performance and lead outcomes.

**Results:**

A total of 498 patients were enrolled, comprising 387 patients with RVP and 111 patients with CSP, with a follow-up duration of 44.42 ± 10.41 months. In patients without a prior history of AF, CSP was associated with a significantly lower incidence of new-onset AHREs when the percentage of ventricular pacing was ≥20% (9.52% vs. 29.70%, *P* = 0.001). After adjusting for confounding factors, CSP exhibited a lower hazard ratio for new-onset AHREs compared to RVP (HR 0.336; [95% CI: 0.142–0.795]; *P* = 0.013), alongside left atrial diameter (LAD) (HR 1.109; [95% CI: 1.048–1.173]; *P* < 0.001). In patients with a history of AF, the progression of AHREs in CSP and RVP did not differ significantly (32.35% vs. 34.75%, *P* = 0.791). Cardiac performance metrics, including left ventricular end-diastolic diameter (LVEDD) (49.09 ± 4.28 mm vs. 48.08 ± 4.72 mm; *P* = 0.015), LAD (40.68 ± 5.49 mm vs. 39.47 ± 5.24 mm; *P* = 0.001), and NYHA class (2.31 ± 0.46 vs. 1.59 ± 0.73; *P* < 0.001), improved obviously following CSP, while LVEDD (48.37 ± 4.57 mm vs. 49.30 ± 5.32 mm; *P* < 0.001), LAD (39.77 ± 4.58 mm vs. 40.83 ± 4.80 mm; *P* < 0.001), NYHA class (2.24 ± 0.43 vs. 2.35 ± 0.83; *P* = 0.018), and left ventricular ejection fraction (LVEF) (57.41 ± 2.42 vs. 54.24 ± 6.65; *P* < 0.001) deteriorated after RVP.

**Conclusion:**

Our findings suggest that CSP may be associated with improvements in cardiac performance and a reduction in new-onset AHREs compared to RVP in patients with HFpEF. However, prospective randomized trials are anticipated to confirm these potential benefits.

## Introduction

Atrial fibrillation (AF) is prevalent among patients with heart failure with preserved ejection fraction (HFpEF), with prevalence rate ranging from 15% to 41%, this condition is associated with an increased risk of hospitalization and elevated mortality rates (Gierula et al., 2022; Sartipy et al., 2019). Unfortunately, right ventricular pacing (RVP) has been shown to exacerbate the heart failure and increase occurrence of AF in patients with a high percentage of ventricular pacing, as demonstrated in previous randomized controlled trials (Sweeney et al., 2003; Sweeney et al., 2007).

Conduction system pacing (CSP) has emerged as a more physiological pacing modality that facilitates cardiac electrical resynchronization compared to RVP. Recent data indicated that both his bundle pacing (HBP) and left bundle branch pacing (LBBP) were associated with a lower incidence of new-onset AF compared to RVP (Ravi et al., 2020; Zhu et al., 2023). Atrial high-rate episodes (AHREs) can be continuously monitored by implanted cardiac devices, are strongly linked to the development of clinical AF and stroke (Toennis et al., 2023; Gonzalez et al., 2014). However, it remains unclear whether CSP would reduce the occurrence of AHREs in patients with HFpEF. This study aims to illustrate the incidence of new-onset AHREs and progressive AF following different pacing modalities in patients with HFpEF, while also exploring improvements in cardiac performance.

## Methods

### Patient enrollment

Patients with HFpEF who had indications for a dual-chamber pacemaker due to atrioventricular conduction block (AVB) were retrospectively and consecutively enrolled at our center from January 2018 to January 2023. Exclusion criteria included atrial lead rupture or unreliable atrial signal detection, loss to follow-up, severe valvular disease (mitral or aortic regurgitation/stenosis of severe grade), heart surgery within the 6 months prior to implantation, a known history of persistent or permanent AF, a history of an atrioventricular node ablation and device upgrades or generator replacements.

### Pacemaker implantation procedure

CSP was performed using lead 3,830 (Medtronic, Minneapolis, MN). His bundle electrogram and left bundle branch electrogram were recorded in a unipolar configuration (Prucka Cardiolab, GE Healthcare, Waukesha, WI, United States). HBP was not considered if 1:1 His–ventricular conduction was not detected while pacing at a rate of 120 beats per minute or patients with infranodal AVB. The unipolar configuration and pacing impedance were monitored alongside the left ventricular activation time (LVAT) (Zhang et al., 2023). Stim-left ventricular active time (LVAT) less than 75 ms, an abrupt decrease in LVAT of longer than 10 ms and the morphologies of Qr, qR, or rSR’ in lead V_1_ were the simple criteria for left bundle branch capture.

### Data collection and patients follow-up

All patients were followed up at 1, 3, 6, and then every 6 months after the procedure. The 12-lead ECG, atrial high-rate episode burden (AHREs), echocardiographic parameters, comorbidities, and medications were documented. The pacing percentage and AHREs were noted at the initial 1-month visit and at each subsequent device interrogation, including remote interrogations. Anticoagulation therapy was recommended according to the guidelines established. The sizes of the left ventricle (LV) and left atrium (LA), as well as cardiac function, were monitored annually via cardiac ultrasound (video, GE Healthcare). The left ventricular ejection fraction (LVEF) was calculated using the modified Simpson method. The incidence of progressive AF and new-onset AHREs was noted and compared between the CSP and RVP pacing modalities.

### Definitions and criteria

AHREs were defined as episodes characterized by an atrial rate of 200 beats per minute or greater, lasting for a minimum of 5 min. The burden of AHREs was quantified as the average percentage of total AHREs occurring throughout the entire follow-up period (Healey et al., 2012). New-onset AHREs were identified as those detected in patients without a prior history of AF before the procedure (Kohno et al., 2011; Minamiguchi et al., 2012). Atrial fibrillation progression was defined as an absolute increase of 10% or more in the average AHREs burden compared to the initial assessment conducted 1 month after device implantation (Ravi et al., 2020). HFpEF was characterized by objective evidence of cardiac structural and/or functional abnormalities indicative of left ventricular (LV) diastolic dysfunction or elevated LV filling pressures, including elevated levels of natriuretic peptides (McDonagh et al., 2021).

### Statistical analysis

Continuous variables exhibiting a normal distribution were reported as mean ± standard deviation and analyzed using a t-test. For non-normally distributed variables, data were expressed as medians with interquartile ranges, and the Mann-Whitney U test was employed. Categorical variables were compared using either the Chi-square test or Fisher’s exact test, with results reported as percentages. Survival curves were estimated using the Kaplan–Meier method. Univariate and multivariable Cox proportional hazard models were utilized to identify predictors of AHREs, where univariate predictors with a P value of less than 0.05 were incorporated into the multivariate Cox proportional hazard model. All statistical analyses were performed using SPSS Version 26.0, with a significance threshold set at P < 0.05.

## Results

### Patient population characteristics

**FIGURE 1:**
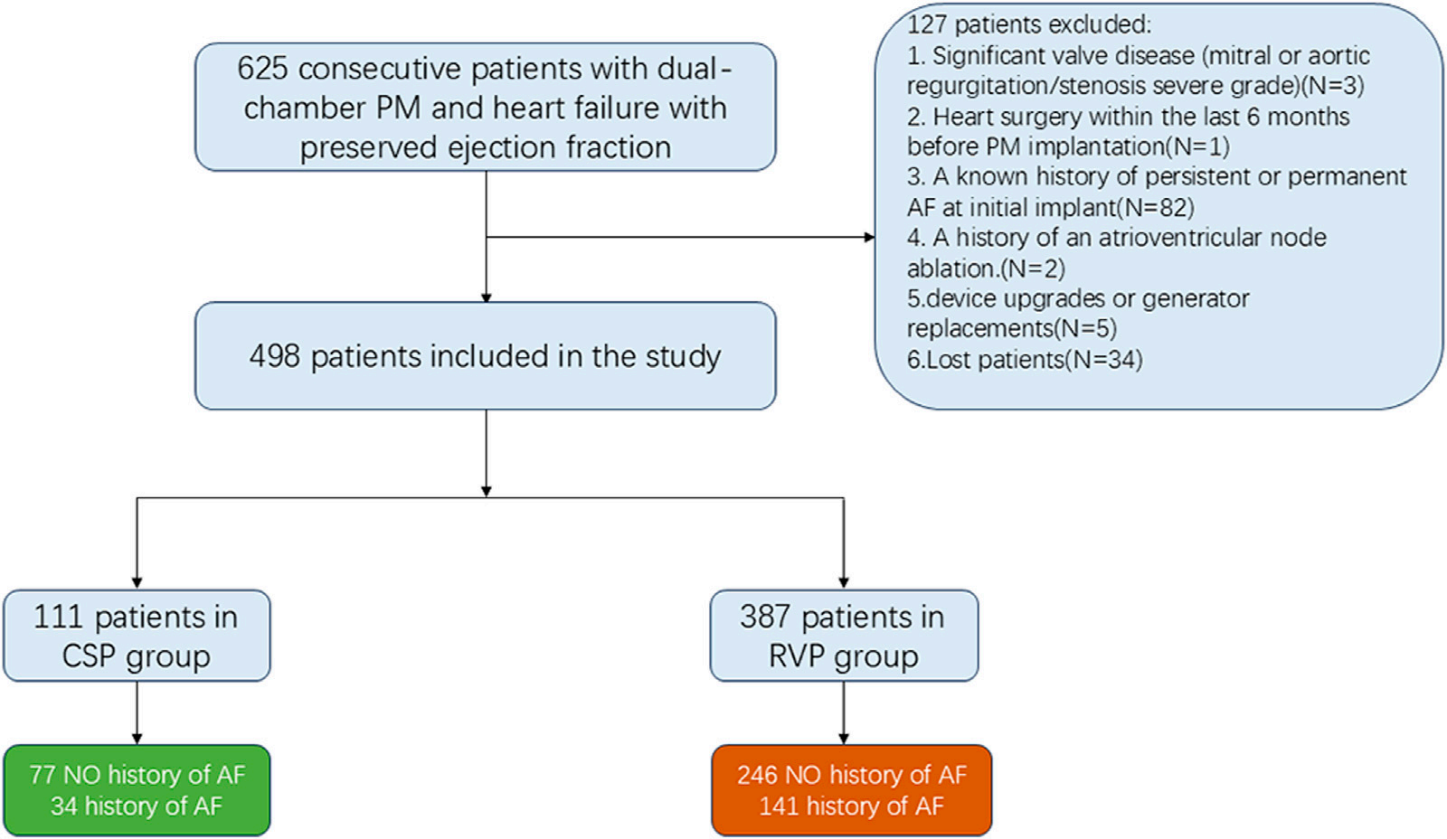
Flowchart showing the patients included in and excluded from the study. CSP: conduction system pacing; RVP: right ventricular pacing; AF: atrial fibrillation; PM: pacemaker.

A total of 625 patients (mean age 73.21 ± 10.00 years; 45.4% male) diagnosed with HFpEF and implanted with dual-chamber pacemakers for AVB were continuously screened from January 2018 to January 2023. Out of these, 498 patients (387 with RVP and 111 with CSP) were enrolled, as illustrated in Figure 1. Among the CSP group, 21 patients (18.92%) received HBP and 90 patients (81.08%) received LBBP. The average follow-up duration was 44.42 ± 10.41 months.

The baseline clinical characteristics of the study population are detailed in Table 1. No statistically significant differences were observed in age, gender, comorbidities, medication usage, left ventricular end-diastolic diameter (LVEDD), left atrial diameter (LAD), left ventricular ejection fraction (LVEF), or New York Heart Association (NYHA) class between the patients receiving CSP and those receiving RVP (all *P* > 0.05).

**TABLE 1:** Baseline characteristics of all patients.

	Patients without history of AF			Patients with history of AF		
	CSP(N = 77)	RVP(N = 246)	*P*-Value	CSP(N = 34)	RVP(N = 141)	*P*-Value
Age, years	71.19 ± 9.96	73.81 ± 10.34	0.051	71.21 ± 7.19	74.06 ± 9.08	0.089
Male, n (%)	38 (49.35)	113 (45.93)	0.600	19 (55.88)	54 (38.30)	0.062
BMI, kg/m^2^	30.72 ± 0.73	30.59 ± 1.71	0.350	27.73 ± 3.52	28.71 ± 3.84	0.184
NYHA class			0.079			0.638
NYHA II, n (%)	49 (63.64)	182 (73.98)		28 (82.35)	111 (78.72)	
NYHA III, n (%)	28 (36.36)	64 (26.02)		6 (17.65)	30 (21.28)	
SND	25 (32.47)	57 (23.17)	0.102	23 (67.65)	105 (74.47)	0.421
Hypertension, n (%)	69 (89.60)	218 (88.60)	0.809	29 (85.29)	115 (81.56)	0.609
CAD, n (%)	7 (9.09)	39 (15.85)	0.138	10 (29.41)	33 (23.40)	0.465
DM, n (%)	18 (23.38)	80 (32.52)	0.128	13 (38.24)	33 (23.40)	0.078
CKD, n (%)	4 (5.19)	22 (8.94)	0.291	5 (14.71)	6 (4.26)	0.063
LVEDD, mm	49.60 ± 4.62	48.88 ± 4.35	0.218	47.94 ± 3.10	47.46 ± 4.83	0.588
LAD, mm	40.15 ± 4.79	39.34 ± 4.14	0.158	41.91 ± 6.74	40.53 ± 5.20	0.198
LVEF, %	57.57 ± 2.01	57.50 ± 2.48	0.806	56.73 ± 2.48	57.27 ± 2.29	0.231
E/e’	17.73 ± 2.54	17.37 ± 2.17	0.221	18.21 ± 4.54	17.37 ± 2.75	0.171
ACEI/ARB/ARNI, n (%)	23 (29.87)	79 (32.11)	0.712	10 (29.41)	48 (34.04)	0.607
AAD, n (%)	6 (7.79)	20 (8.13)	0.924	6 (17.65)	22 (15.27)	0.770
AF ablation	—	—	—	3 (8.82)	20 (14.18)	0.584
BNP, pg/mL	188.00 (152.50,392.86)	227.00 (154.92,393.44)	0.533	201.23 (152.50,374.35)	233.35 (158.23,446.73)	0.612

BMI: body mass index; SND: sinus node disease; CAD: coronary artery disease; DM: diabetes mellitus; CKD: chronic kidney disease; LVEDD: left ventricular end-diastolic diameter; LAD: left atrial diameter; LVEF: left ventricular ejection fraction; ARNI: angiotensin receptor-neprilysin inhibitors; ACEI: angiotensin-converting enzyme inhibitors; ARB: angiotensin receptor blockers; AAD: antiarrhythmic drug; CSP: conduction system pacing; RVP: right ventricular pacing; AF: atrial fibrillation.

### Procedure outcomes in different pacing modalities

The paced QRS duration was significantly shorter in CSP compared to RVP (116.86 ± 23.75 ms vs. 144.53 ± 32.00 ms, *P* < 0.001). The capture threshold was higher in the CSP than in RVP (1.05 ± 0.31 V vs. 0.83 ± 0.18 V, *P* < 0.001). An increase in capture threshold of ≥2 V at 0.4 ms was observed in 4 patients with CSP and 2 patients with RVP (3.60% vs. 0.52%, *P* = 0.033). The incidence of procedural complications was similar between the CSP and RVP groups (0.90% vs. 0.26%, *P* = 0.926). Details of the procedures were shown in Table 2.

**TABLE 2:** Procedures and clinical outcomes.

	CSP (N = 111)	RVP (N = 387)	*P*-Value
Baseline QRS duration, ms	108.20 ± 27.57	107.84 ± 31.90	0.914
Paced QRS duration, ms	116.86 ± 23.75	144.53 ± 32.00	<0.001
Threshold, V	1.05 ± 0.31	0.83 ± 0.18	<0.001
Threshold increase≥2 V, n (%)	4 (3.60)	2 (0.52)	0.033
Procedural complications	1 (0.90)	1 (0.26)	0.926
Pericardial effusion	0	0	
Pneumothorax	1	0	
Lead dislodgement	0	1	
Infection	0	0	
VP%≥20%, n (%)	80 (72.07)	256 (66.15)	0.240

VP%: percentage of ventricular pacing; CSP: conduction system pacing; RVP: right ventricular pacing.

### Clinical outcomes in different pacing modalities

Clinical outcomes in different pacing modalities demonstrated significant differences. Patients with RVP experienced deterioration in NYHA class (2.24 ± 0.43 vs. 2.35 ± 0.83, *P* = 0.018), LVEDD (48.37 ± 4.57 mm vs. 49.30 ± 5.32 mm, *P* < 0.001), LAD (39.77 ± 4.58 mm vs. 40.83 ± 4.80 mm, P< 0.001), and LVEF (57.41% ± 2.42% vs. 54.24% ± 6.65%, *P* < 0.001) after follow-up. In contrast, patients with CSP showed improvement in NYHA class (2.31 ± 0.46 vs. 1.59 ± 0.73, *P* < 0.001), LVEDD (49.09 ± 4.28 mm vs. 48.08 ± 4.72 mm, *P* = 0.015), and LAD (40.68 ± 5.49 mm vs. 39.47 ± 5.24 mm, *P* = 0.001), as illustrated in Table 3.

**TABLE 3:** Clinical outcomes comparation in different pacing modalities.

	Baseline	After follow-up	*P*-Value
NYHA class in CSP	2.31 ± 0.46	1.59 ± 0.73	<0.001
NYHA class in RVP	2.24 ± 0.43	2.35 ± 0.83	0.018
LAD in CSP, mm	40.68 ± 5.49	39.47 ± 5.24	0.001
LAD in RVP, mm	39.77 ± 4.58	40.83 ± 4.80	<0.001
LVEDD in CSP, mm	49.09 ± 4.28	48.08 ± 4.72	0.015
LVEDD in RVP, mm	48.37 ± 4.57	49.30 ± 5.32	<0.001
LVEF in CSP, %	57.32 ± 2.18	56.57 ± 4.96	0.121
LVEF in RVP, %	57.41 ± 2.42	54.24 ± 6.65	<0.001

LAD: left atrial diameter; LVEDD: left ventricular end-diastolic diameter; LVEF: left ventricular ejection fraction; CSP: conduction system pacing; RVP: right ventricular pacing.

### New-onset AHREs in different pacing modalities

**FIGURE 2:**
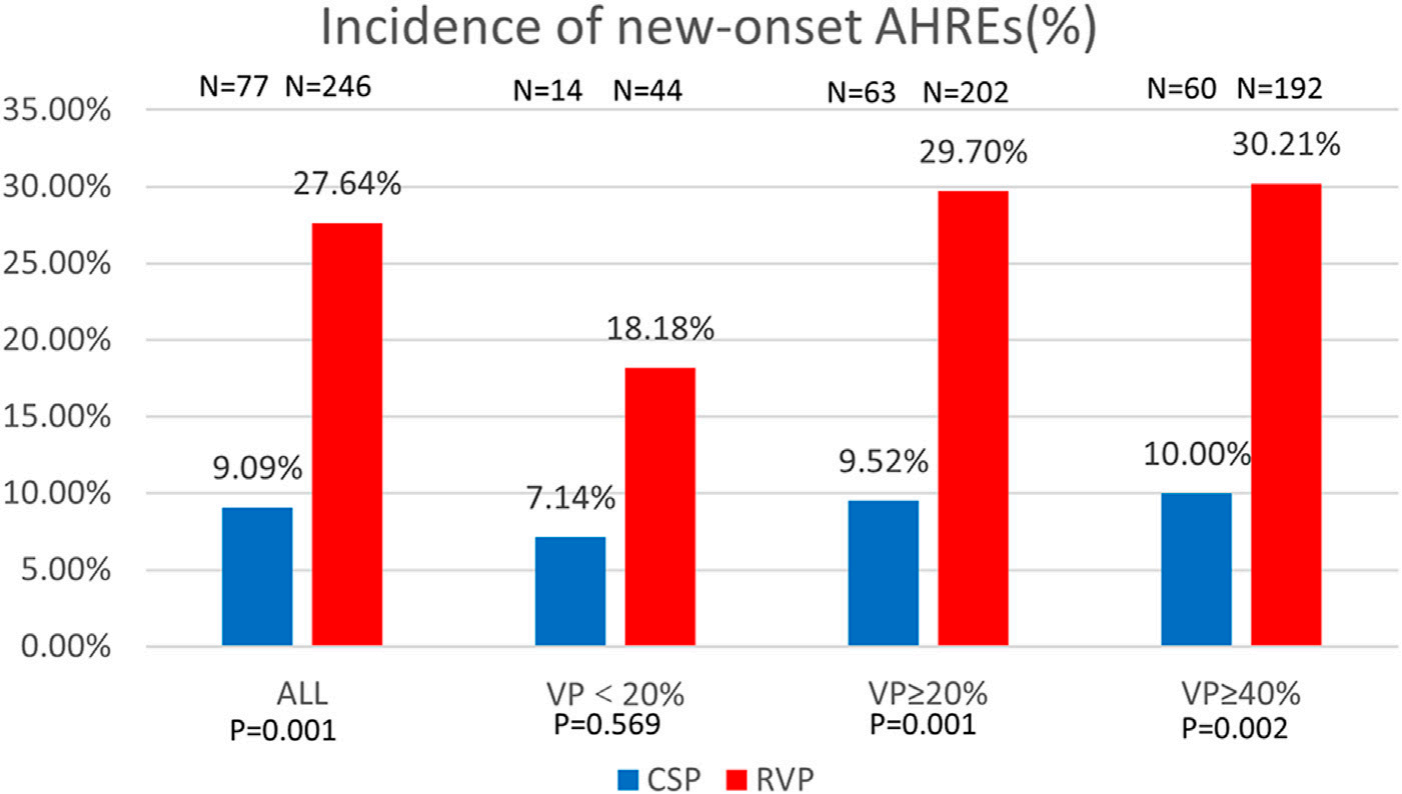
Comparison of new-onset AHREs by percentage between CSP and RVP in all patients and the subgroups based on ventricular pacing (%). AHREs: atrial high-rate episodes; VP: ventricular pacing.

**FIGURE 3:**
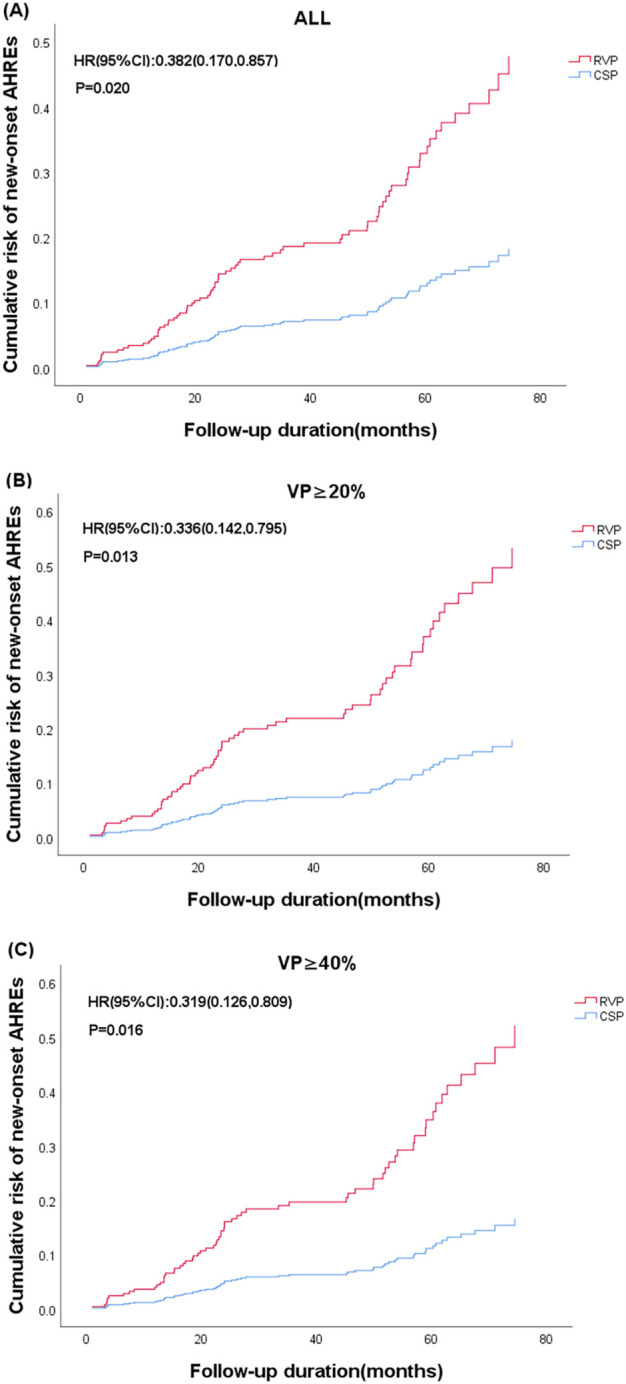
Cumulative risk of new-onset AHREs based on the type of device (CSP vs. RVP) and subgroups stratified by ventricular pacing (%). **(A)** All enrolled patients; **(B)** Ventricular pacing (VP)≥20%; **(C)** Ventricular pacing (VP)≥40%. The representations derived from stratified multifactorial Cox model risk function. The P values from Cox proportional risk model. CSP: Conduction system pacing; RVP: right ventricular pacing; HR: hazard ratio.

Regarding new-onset AHREs in different pacing modalities, 77 patients with CSP and 246 patients with RVP had no prior history of AF before the procedure. New-onset AHREs were identified in 7 patients (7/77, 9.09%) with CSP and 68 patients (68/246, 27.64%) with RVP (*P* = 0.001), as shown in Figure 2. CSP was associated with a significantly lower incidence of new-onset AHREs compared to RVP, particularly in patients with a ventricular pacing percentage of ≥20% (9.52% vs. 29.70%, *P* = 0.001), while no significant difference was observed in those with a ventricular pacing percentage of less than 20%, as depicted in Figure 2. Univariate and multivariate Cox regression models were employed to identify predictors of new-onset AHREs, as presented in Table 4. CSP exhibited a lower hazard ratio for new-onset AHREs compared to RVP (HR 0.336; [95% CI: 0.142–0.795]; *P* = 0.013), as well as LAD (HR 1.109; [95% CI: 1.048–1.173]; *P* < 0.001). The cumulative risks of new-onset AHREs across different pacing modalities are illustrated in Figure 3.

**TABLE 4:** Cox regression analysis for risk factors of new-onset AHREs in patients without history of AF.

	Patients with VP ≥ 20%					
	Univariate analysis			Multivariate analysis		
	HR	95% CI	*P*-Value	HR	95% CI	*P*-Value
CSP	0.396	0.169–0.923	0.032	0.336	0.142–0.795	0.013
Age	1.018	0.993–1.044	0.156			
Male	1.245	0.768–2.018	0.374			
BMI	1.106	0.893–1.369	0.357			
SND	1.195	0.727–1.965	0.483			
Hypertension	0.794	0.415–1.520	0.487			
DM	0.867	0.505–1.491	0.607			
CAD	1.008	0.499–2.036	0.983			
AAD	2.494	0.998–6.233	0.050			
ACEI/ARB/ARNI	1.186	0.710–1.980	0.515			
LAD	1.091	1.036–1.150	0.001	1.109	1.048–1.173	<0.001
LVEDD	1.009	0.953–1.068	0.759			
LVEF	1.016	0.924–1.117	0.745			
AP%	1.005	0.993–1.018	0.409			

BMI: body mass index; SND: sinus node disease; DM: diabetes mellitus; CAD: coronary artery disease; AAD: antiarrhythmic drug; ACEI: angiotensin-converting enzyme inhibitors; ARB: angiotensin receptor blockers; ARNI: angiotensin receptor-neprilysin inhibitors; LAD: left atrial diameter; LVEDD: left ventricular end-diastolic diameter; LVEF: left ventricular ejection fraction; CSP: conduction system pacing; RVP: right ventricular pacing; AP%: percentage of atrial pacing; AHREs: atrial high-rate episodes.

## Progression of AHREs in different pacing modalities

**FIGURE 4:**
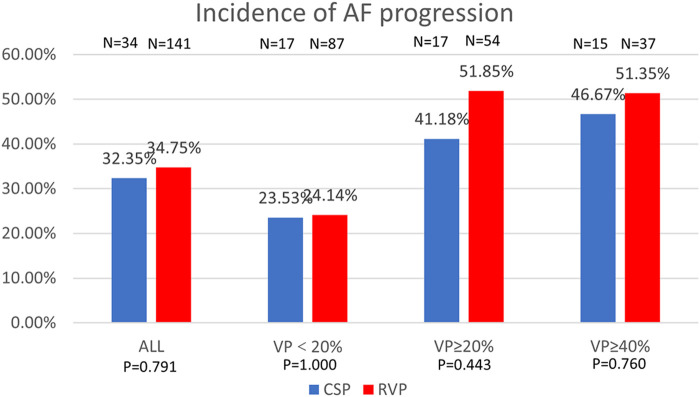
Comparison of AF progression by percentage between CSP and RVP in all patients and the subgroups based on ventricular pacing (%). AF: atrial fibrillation; VP: ventricular pacing.

In terms of the progression of AF in different pacing modalities, 34 patients with CSP and 141 patients with RVP had paroxysmal AF prior to pacemaker implantation. Progression of AF occurred in 11 patients (11/34, 32.35%) with CSP and 49 patients (49/141, 34.75%) with RVP (*P* = 0.791). The progression of AF was comparable between CSP and RVP, regardless of the ventricular pacing percentage, as shown in Figure 4.

## Discussion

### Main findings

This study first demonstrates that CSP might be effective than RVP in improving cardiac performance and reducing AHREs in patients with HFpEF. Furthermore, it suggests CSP and LAD are independent predictors of new-onset AHREs.

### Pacing modalities on cardiac performances in patients with HFpEF

Current randomized trials have demonstrated the superiority of biventricular pacing (BiVP) over RVP in enhancing quality of life, NYHA class, and echocardiographic outcomes in patients with moderate to severe systolic dysfunction (Filho et al., 2010; Curtis et al., 2013; Kindermann et al., 2006). However, the high cost and limited response of BiVP rendered it inaccessible for some patients. Zhang et al. demonstrated that CSP significantly improve NYHA class, LVEF, and LVEDD in patients with HFmrEF and a high percentage of ventricular pacing (Zhang et al., 2023). HFpEF is characterized by increasing rates of hospitalization and mortality, highlighting the need for new therapeutic options (Infeld et al., 2023).

CSP has been recommended as an alternative to RVP in patients with AVB (Chung et al., 2023). Reports indicated that LBBP resulted in greater improvement in BNP levels compared to RVP (O et al., 2022). Moreover, HBP has been shown to improve NYHA class and reduce diuretic use in HFpEF patients after 1 year (Huang et al., 2017). However, data regarding the long-term impact of CSP on cardiac remodeling in HFpEF remained scarce. Consistent with previous studies, this research also demonstrated favorable cardiac function, including improvements in LVEF and NYHA class following CSP. Additionally, it explored the benefits of CSP on cardiac reverse remodeling, revealing significant improvements in LVEDD (P = 0.015) and LAD (P = 0.001) after long-term follow-up. In contrast, RVP was associated with deterioration in LVEDD (P < 0.001), LAD (P< 0.001), NYHA class (P = 0.018), and LVEF (P< 0.001). These positive outcomes might result from a combination of factors, and the individual atrioventricular interval optimization could play a role except for the physiological electrical conduction facilitated by CSP (Cobb and Gold, 2017).

### Predictors of new-onset AHREs in patients with HFpEF

AF was prevalent among patients with a significant proportion of ventricular pacing (Sweeney et al., 2003). Recent studies have demonstrated that the incidence of AHREs in patients with RVP was approximately 2.3 times greater than those with LBBP (Takahashi et al., 2023; Zhang et al., 2024). In line with these observations, our analysis revealed that CSP was associated with a reduced incidence of AHREs (HR 0.336, 95% CI: 0.142–0.795) after adjusting for confounding variables using multifactorial regression analysis. Notably, the prevalence of AHREs was found to be as high as 29.70% in patients with RVP of VP%≥20%, compared to only 9.52% in those receiving CSP (P = 0.001).

Structural alterations in chronic heart failure patients, compounded by neurohormonal activation, significantly increased the prevalence of AF (Kotecha and Piccini, 2015). HFpEF was primarily characterized by left ventricular concentric remodeling, hypertrophy, and diastolic dysfunction (CS et al., 2007). Additionally, left atrial enlargement, along with cardiac volume and pressure overload, has been shown to correlate with the occurrence of AF (Hoit, 2014). Study has confirmed that left atrial enlargement is a significant risk factor for AHREs (Kim et al., 2021). However, this cohort study is the first to demonstrate that left atrial enlargement is associated with the development of AHREs in patients with HFpEF. In patients with HFpEF, left atrial enlargement served as a well-established proarrhythmic substrate associated with atrial fibrosis (Knackstedt et al., 2008). Importantly, our findings explored that CSP contributed to left atrial remodeling (40.68 ± 5.49 vs. 39.47 ± 5.24, *P* = 0.001) when compared to RVP (39.77 ± 4.58 vs. 40.83 ± 4.80, *P* < 0.001), which might play a role in the prevention of AF.

Previous studies had demonstrated that the risks of ventricular desynchrony, adverse remodeling, and atrial arrhythmia increased when the percentage of ventricular pacing exceeded 20%–40% (Sweeney et al., 2003; Ravi et al., 2020; Kiehl et al., 2016; Khurshid et al., 2014).Additionally, several studies had established a correlation between elevated atrial pacing percentages and an increased risk of atrial arrhythmias (Bukari et al., 2018; Fontenla et al., 2016; Biffi et al., 2019; Ziacchi et al., 2018). However, the population enrolled in this study primarily consisted of patients with atrioventricular block, and the atrial pacing percentages within this cohort limited its ability to predict the incidence of AHREs.

### Different pacing modalities on AHREs in patients with HFpEF

Current data suggested that HBP was associated with a reduced risk of AHREs compared to RVP in patients without AF history (Takahashi et al., 2023). However, the extent to which the physiological advantages of CSP in preventing AHREs could be counterbalanced by the effects of heart failure in patients with HFpEF remains unclear. Our study first confirmed the benefits of CSP in reducing new-onset AHREs even among patients with HFpEF. However, in the patients with a prior history of AF, no statistically significant differences were observed in the progression of AHREs between CSP and RVP. Additionally, Pastore et al. found no difference in the progression of AF between HBP and RVP (Pastore et al., 2020). These findings underscore the necessity of early AF management and the importance of primary prevention. It was noteworthy that a higher proportion of patients with a history of AF in the RVP group had previously undergone AF ablation compared to those in the CSP group (14% vs. 8%). This disparity might diminish the potential benefits of CSP in reducing AF burden among patients who had already undergone ablation.

RVP-related diastolic dysfunction, asynchronous mitral regurgitation and enlarged left atrial expansion increased the risk of AF occurrence (Naqvi and Chao, 2023). CSP would be beneficial in reducing the incidence of AF by enhancing the cardiac electrical and mechanical synchronization, as well as delaying left atrial reverse remodeling (Cai et al., 2020; Hou et al., 2019). HFpEF was associated with progressive left atrial myopathy which served as the substrate of AF occurrence (Kotecha et al., 2016). Consequently AF recurrence was more prevalent in patients with HFpEF regardless of the type of AF (Younis et al., 2024). These findings suggested that managing AF in patients with concomitant heart failure presented significant challenges, particularly for those with a prior history of AF. Merely altering the pacing modality without addressing multiple risk factors is unlikely to effectively prevent AF recurrence. Therefore, the management of various risk factors associated with AF, such as diabetes mellitus, obesity, alcohol consumption, and sleep apnea syndrome, should be considered essential for the prevention of AF.

Although the age difference between the CSP and RVP did not reach statistical significance (*P* = 0.051), we agreed that younger patients generally present a lower risk of atrial arrhythmias. Notably, all enrolled patients in this study were elderly, which somewhat diminished the influence of age on the final results. And it was further demonstrated that age was not identified as a statistically significant factor associated with the incidence of AHREs in the univariate regression analyses (*P* = 0.156).

## Limitations

This study was a single-center, retrospective observational analysis. Given the potential for selection bias associated with its non-randomized design, caution should be exercised when interpreting the results. A randomized multi-center trial with a larger sample size may be necessary to validate these findings.

## Conclusion

Our findings suggest that CSP may be associated with improvements in cardiac performance and a reduction in new-onset AHREs compared to RVP in patients with HFpEF. However, prospective randomized trials are anticipated to confirm these potential benefits.

## Data Availability

The raw data supporting the conclusions of this article will be made available by the authors, without undue reservation.
